# Interferon Regulatory Factor 3 Deficiency Induces Age-Related Alterations of the Retina in Young and Old Mice

**DOI:** 10.3389/fncel.2019.00272

**Published:** 2019-06-20

**Authors:** Xi Zhang, Jingyi Zhu, Xianjun Chen, Zhang Jie-Qiong, Xue Li, Linlin Luo, Huang Huang, Wenyi Liu, Xinyuan Zhou, Jun Yan, Sen Lin, Jian Ye

**Affiliations:** ^1^Department of Ophthalmology, Institute of Surgery Research, Army Medical Center of PLA (Daping Hospital), Army Medical University, Chongqing, China; ^2^Department of Histology and Embryology, Chongqing Key Laboratory of Neurobiology, Army Medical University, Chongqing, China; ^3^Institute of Immunology, Army Medical University, Chongqing, China

**Keywords:** *IRF3*, retina, ERG, retinal thickness, synaptic ectopia, senescence

## Abstract

Age-related changes in visual function and retina structure are very common in aged animals, but the underlying mechanisms of these changes remain unclear. Here we report that the expression of interferon regulatory factor 3 (*IRF3*), a critical immune regulatory factor, is dramatically down-regulated in mouse retinas during aging. To address the role of *IRF3* in the retina, we examined the structure and function of retinas in young (3–4 months) and old (22–24 months) *Irf3*^-/-^ mice in comparison to age-matched wildtype (WT) mice. We found that *IRF3* deletion resulted in impaired electroretinogram (ERG) responses and decreased retinal thickness in both young and old mice. In addition, numerous synapses of the outer plexiform layer (OPL) were found obviously extending into outer nuclear layer (ONL) in *Irf3*^-/-^ mice, along with a reduction of the average synapse density in the OPL. These changes suggest that *IRF3* deletion may accelerate retinal senescence. In support of this hypothesis, a number of classic senescence-associated markers were found in remarkably elevated level in *Irf3*^-/-^ retina, including p53, p16^INK4a^, inositol-requiring enzyme 1α (IREα), p-H2A.X and promyelocytic leukemia protein (PML). Overall, our results indicate that maintenance normal *IRF3* levels is necessary for retinal structure and function and suggest that *IRF3* is an important regulator of retinal senescence.

## Introduction

A gradual decline of retinal functions has been well-recognized during aging (Jackson and Owsley, 2003; Andersen, 2012; Boya et al., 2016). Architectural disruptions in the retina have been found to be important indicators of functional decline, as retinal function primarily relies on its elegantly composed structures (Bonnel et al., 2003; Nag and Wadhwa, 2012; Zueva et al., 2016). Numerous studies have shown age-related structural changes that occur in the retina, including reduced retinal thickness (Archibald et al., 2011; Samuel et al., 2011), declining synapse density, and decreased areas of dendritic and axonal arbors in aged mice (Liets et al., 2006). In addition, the horizontal and bipolar neurons extend their dendritic fibers aberrantly beyond the normal OPL boundary into the photoreceptor nuclear layer in aged retina (Eliasieh et al., 2007). Also, the continuous loss of photoreceptors, the attenuation of choroidal thickness, and the formation of hard drusen in the periphery occur in the retina while aging (Ardeljan and Chan, 2013; Grossniklaus et al., 2013). However, the underlying mechanisms responsible for age-related changes in the retina remain largely unknown.

*IRF3* is a well-known transcriptional factor that participates in regulating the expression of IFN-β and anti-virus responses (Oshiumi et al., 2003; Kanda et al., 2007; Zhao et al., 2011). A recent study has also shown that *IRF3* is involved in DNA-damage associated cell senescence and premature aging by stimulating cell-autonomous IFN-β expression and p53 pathway activation (Yu et al., 2015). It has been previously shown that IRF3 is associated with age-related retinopathy, suggesting that *IRF3* may be important for the structural integrity of retina (Kleinman et al., 2012; Patel and Hackam, 2013, 2014). Therefore, it is important to understand the function of *IRF3* in the retinal aging process.

In this study, we found that the expression of *IRF3* significantly decreases in the retina during aging. Furthermore, we found that *IRF3* deletion disrupted retinal function as revealed by electroretinogram (ERG). In addition, *IRF3* deficiency also caused morphological changes of the retina, including decreased retinal thickness, average density reduction and ectopia of the OPL synapse. Those changes are reminiscent of the phenotypes detected in aged mice. Consistently, several senescence-related markers were greatly up-regulated in the retina of *Irf3*^-/-^ mice compared with WT mice of the same age. Together, our results are the first to reveal that insufficient production of *IRF3* affects the structure and function of the retina and may play an important role in accelerating retinal senescence.

## Materials and Methods

### Mouse Strains

The *Irf3* transgenic mice (stock number RBRC00858, B6;129S6-Bcl2l12/Irf3 < tm2Ttg > /TtgRbrc) were kindly provided by Prof. Zou Qiang in Chengdu Medical College, which were purchased from RIKEN institute, Japan. The full characterization of these mice has been described elsewhere (Prantner et al., 2010, 2011). All mice used in this study were backcrossed (ten generations) to the C57BL/6J strain (Jackson Laboratories). All studies were performed using male mice. To identify the genotype of mice, PCR, real-time RT-PCR and Western blot were used to detect the genome, mRNA and protein level of IRF3. The young adult (YA) mice used in the study are among 3–4 months, the old mice are 22–24 months, were bred in our vivarium. The Institutional Animal Care and Use Committee (IACUC) of the Army Medical University approved all studies using animals.

### Optical Coherence Tomography (OCT)

The abdominal cavity of each mouse was injected with chloral hydrate (430 mg/kg) for anesthesia. The pupils were scattered by a drop of 1% tropicamide. OCT image acquisition and analysis obtained by Heidelberg Spectralis and Bioptigen Envisu system, averaged transverse scan images were used as a measure of retinal thickness (Zhou et al., 2018). Measurements were applied on four areas of each eye (0.5 mm from optic disk at vertical and horizontal directions), and the average of the four measurements is considered to be the retinal thickness of eye. The total retinal thickness was calculated from the distance between internal limiting membrane (ILM) and Bruch’s membrane (BM). Data are presented as mean ± SEM.

### Histological Examination

Briefly, mice were killed with overdose CO2, eyes were embedded in Tissue Freezing Medium (Sakura Tissue-Tek O.C.T) at 4°C for 10 min, -80°C for 30 min, and sectioned at 10 μm in a cryostat. Sections were fixed with 4% paraformaldehyde (PFA) for 15 min, washed with PBS. HE staining (Solarbio) was used to observe the structure of the retina. Sections were examined with a LEICA DM1000 microscope. 10× and 40× objective was used for histological measurements. The images were captured by LAS V48 system and were then further processed with Image J. Twelve tissue blocks from six mice (two blocks per mouse) were taken from each group. Three areas of the retina (central, media, periphery) were included in the measurements (2∼3 measurements per tissue bloc). The following parameters were measured: (i) The thickness of retinal cross-section from outer limiting membrane to the inner limiting membrane (OLM-ILM); (ii) the width of each layer of retina.

### ERG Examination

Animals were adapted to darkness overnight (>12 h) in advance and all operations were performed in dim red light. Each mouse was injected with a mixture of ketamine (60 mg/kg) and xylazine (9 mg/kg) in the abdominal cavity. The body temperature was maintained at 37°C by a warm heating pad. Mydriasis was achieved by a drop of 1% tropicamide. Flash-induced ERG of both eyes was recorded simultaneously using RetiMINER 3.0 system (IRC, China). Stimulus flashes were produced with flash stimulator (Light Burst). Stimulus intensities increased from -2.0 to 1.0 log cd^∗^s/m2 in 0.5-log unit steps were used in the dark. A 1-min interval was presented between continuous light stimuli.

### Immunostaining

Mice were anesthetized as mentioned earlier, and then eyes were fixed with 4% paraformaldehyde (PFA) for 30 mins, the lens and cornea were removed, and the optic cup was post fixed with 4% PFA for 2 h, followed by 30% sucrose dehydration at 4°C overnight. Optic cup was embedded in Tissue Freezing Medium (Sakura Tissue-Tek O.C.T), sectioned at 10 μm in a cryostat. Sections were incubated with blocking solution for 1 h at room temperature. The blocking solution is made of 1.5% BSA and 3% donkey serum, with 0.3% Triton X-100 in PBS. Primary antibodies were diluted in blocking solution for overnight incubation at 4°C, after 3 times rinse in phosphate buffer saline (PBS), sections with fluorophore-labeled secondary antibodies (Thermo scientific) were incubated at room temperature for 2 h. And the nucleus was stained with DAPI for 5 min. Retinas from 4–6 mice were analyzed in each experimental group. The primary antibodies applied in immunofluorescence were: Bassoon (1:400, Enzo Life Sciences), IRF3 (1:400, Abcam), calbindin (1:1000, Abcam), PKCα (1:500, anti-mouse, Abcam), PSD-95 (1:400, Millipore), P-H2A.X (1:1000, Cell Signaling Technology), syntaxin I (1:500, anti-mouse, Abcam), Sox9 (1:500 Millipore). The sections after the staining were mounted with DPX (Sigma) or Vectashield (Vector Laboratories). Sections were captured by the confocal microscope (Leica SP8) and the corresponding images were collected by Leica Application Suite X. Series of images were overlaid to a z-projection with maximum intensities. The analysis of retinal micrographs was taken with ImageJ and the data was analyzed by GraphPad Prism 6.0.

### Immunoblotting and Analysis

Mice were sacrificed by inhalation of CO2, and eyes were removed to obtain the whole retina. Retinas (*n* = 6 in each group) were ground in RIPA lysis buffer (Thermo scientific) with 1 mM PMSF and 2 mM EDTA. The lysate was centrifuged at 12,000 rpm for 15 min at 4°C, and the supernatant was collected. Protein in per sample was 2.5–2.7 mg added with 5× loading buffer for 8 min boiling, loaded on 4–12% SDS-polyacrylamide gels (Beyotime), transferred onto PVDF membranes. The membranes were blocked with 5% non-fat dry milk dissolved in Tris-buffered saline for 2 h at room temperature. Then the membranes were sequentially probed overnight at 4°C with primary antibodies diluted with blocking solution. The primary antibodies were arranged for the following molecules: IRF3 (1:1000, Abcam), P53 (1:1000, Santa Cruz Biotechnology), P16^INK4a^ (1:1000, Proteintech), PML (1:1000, Santa Cruz Biotechnology), P-H2A.X (1:1000, Cell Signaling Technology), IREα (1:1000, Cell Signaling Technology), GAPDH (1:1,000, Invitrogen). Membranes were washed with TBS-T (pH = 7.5) containing 0.2% Tween for three times, and incubated with appropriate secondary antibody for 90 min at room temperature. The band intensities were measured using exposure machine (Omega Lum^TM^ G). Quantification of band intensity was analyzed by using ImageJ and GraphPad Prism 6.0.

### Electron Microscopy

Electron microscopy was performed as previously described (Chen et al., 2015). Mice were killed by an overdose of CO2. The retinas of each group of mice were removed quickly and fixed with fresh fixative (2% glutaraldehyde in 0.1 M cacodylate buffer) overnight at 4°C. Retinas were rinsed in phosphate buffer saline (PBS), post fixed in 1% OsO4 in PBS for 2 h at room temperature, co-stained with uranyl acetate, dehydrated strictly in a graded ethanol series, fully infiltrated with propylene oxide, and slowly embedded in epon. Thin sections were stained with toluidine blue. Ultrathin sections (∼70 nm) were obtained by an ultramicrotome (LKB-V, LKB Produkter AB, Bromma) and were viewed with a transmission electron microscope (TECNAI10, Philips). Sixteen sections from each mouse retina were analyzed and four mice were selected for each group. The position of the OPL layer was observed in each section under 12,000 magnification.

### Statistical Analysis

**FIGURE 1:**
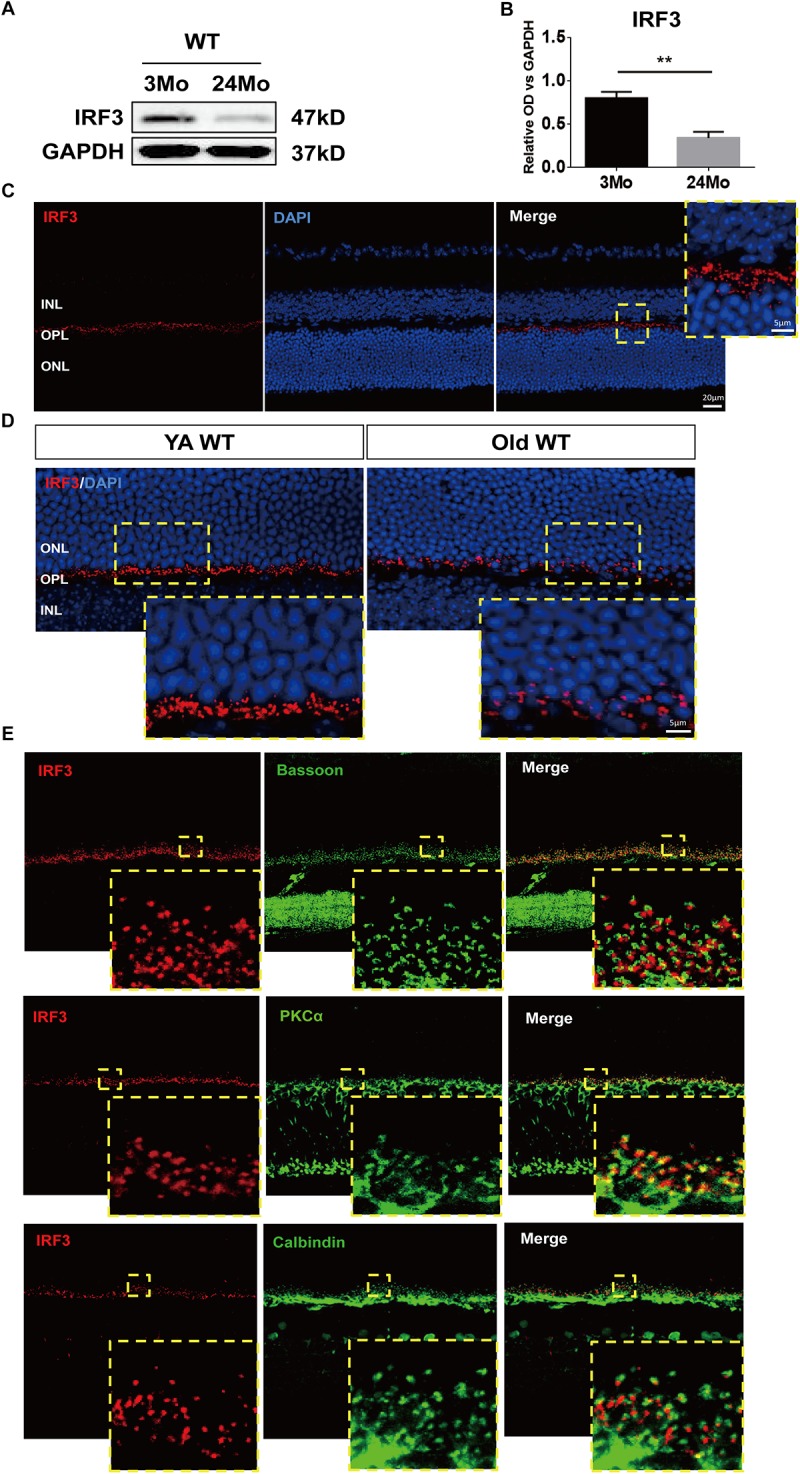
*IRF3* is reduced in mouse retina during aging and expressed in the outer plexiform layer (OPL). **(A)** Representative Western blot band of *IRF* expression in retina in both YA (young adult, 3–4 months) and Old (22–24 months) WT mice. GAPDH served as the loading control. **(B)** Quantification of the expression of *IRF3* at protein levels in YA and old WT mice. Data are presented as mean ± SEM (^∗∗^*p* < 0.01, two-tailed Student’s *t*-test, *n* = 5 per group). **(C)** Immunohistochemical localization of *IRF3* (red) in total retina. Scale bars represents 20 μm. The yellow dotted frame displayed higher-magnification views of the outlined areas (Scale bars represents 5 μm). **(D)** The immunofluorescence staining of *IRF3* was compared in YA and Old WT mice. Cell nuclei was stained by DAPI. Scale bars = 5 μm. **(E)** *IRF3* is mainly localized at the dendritic terminal of rod bipolar cells as revealed by co-labeling with Bassoon (presynaptic protein, green), PKCα (rod bipolar cells, green), and Calbindin (horizonal cells, green).

All analyses were carried out with Prism software version 6 (GraphPad). For two-sample comparisons (Western blot results, Figure 1B), an unpaired, two-tailed Student’s *t*-test was applied. Data in other figures was analyzed with two-way ANOVA followed by either a Bonferroni or Kruskal-Wallis *post hoc* test. All data are presented as mean ±SEM. *P*-values of less than 0.05 (*P* < 0.05) were considered to be statistically significant.

## Results

### *IRF3* Expression in Young and Aged Mouse Retina

In order to explore the role of *IRF3* in retinas, we evaluated the total change of *IRF3* protein by using western blotting from a general perspective. Interestingly, *IRF3* was significantly reduced in old mouse retinas compared with those of young mice (Figure 1A,B). To further ascertain the role that *IRF3* plays in retinas, we uncovered the location of *IRF3* in YA WT mice by staining *IRF3* with DAPI. The results indicated that *IRF3* was expressed in outer plexiform layer (OPL) (Figure 1C). Moreover, consistent with the western blotting results, we noticed that the expression of *IRF3* in the retina of Old WT mice had decreased significantly and that some had entered the ONL by comparing retinal fluorescence staining for YA and Old WT mice (Figure 1D). Furthermore, we found that *IRF3* may be mainly localized at dendritic terminals of rod bipolar cells by labeling with PKCα (Figure 1E). Taken together, the location of IRF3 expression in OPL and the declining *IRF3* expression in aged retina suggest that *IRF3* may play an important role in retinal function.

### *IRF3* Is Necessary for Physiological Function of Retina

**FIGURE 2:**
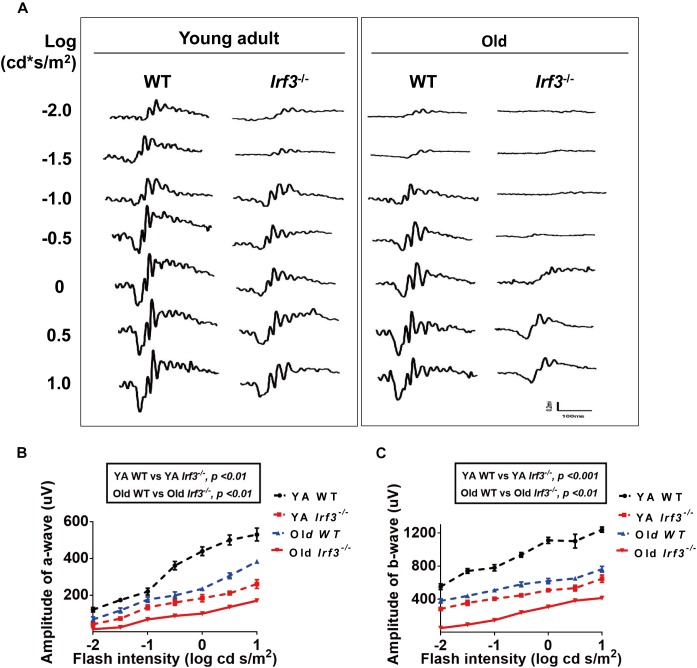
*IRF3* deficiency causes abnormal physiological function of the retina. **(A)** Analysis of ERG a- and b-wave responses in both YA and old WT and *Irf3*^-/-^ mice. Representative scotopic ERG recordings evoked by white light flashes of increasing intensities; traces on the left-side of the figure are shown as the log intensity (log cd s/m^2^). **(B,C)** Quantification of ERG amplitudes in a- and b-waves of dark-adapted mice in each experimental group. a-wave amplitudes **(B)** and b-wave amplitudes **(C)** measured from recordings of dark-adapted YA and old WT and *Irf3*^-/-^ mice. The amplitudes of a- and b-waves in *Irf3*^-/-^ mice were significantly different from those in WT mice of the same age. Data are presented the mean ± SEM (a-wave: *p* < 0.001, b-wave: *p* < 0.001; *n* = 9 per group).

To determine the role that *IRF3* plays in retinal function, we examined the retinas of the young adult (YA) and Old *Irf3*^-/-^ mice and their corresponding control littermates, YA WT and Old WT, via ERG. After flash ERG stimuli, the responses of photoreceptor cells (a-wave) and summed synaptic responses for both inner and outer retina (b-wave) (Samuel et al., 2014) were significantly weakened in *Irf3*^-/-^ mice compared with WT mice of the same age, represented by the lower amplitude of a-wave and b-wave in both YA and Old *Irf3*^-/-^ groups (Figure 2A–C). Interestingly, with increased ERG stimulation intensity, the a-wave and b-wave amplitudes also increased significantly, but the responses in *Irf3*^-/-^ mice were consistently weaker than in WT mice of the same age (Figure 2A–C). As ERG recordings were tested under dark-adapted conditions, the results indicated that the *Irf3*^-/-^ mice largely lacked rod-driven responses. To be noted, PNA (peanut agglutinin) and cone arrestin staining revealed that the deletion of *IRF3* did not cause cone cells loss, which instead resulted from age (Supplementary Figures S1A–C).

These results demonstrate that YA *IRF3*-deficient mice exhibit alterations in retinal physical function similar to those that have been reported for old WT mice (Jackson and Owsley, 2003; Owsley, 2011). In addition, Old *Irf3*^-/-^ mice presented worse retinal physical function than old WT mice, indicating that *IRF3* is crucial for retinal function. However, whether the structure of the retina is affected in *Irf3*^-/-^ mice requires further study.

### *IRF3* Deletion Resulted in Decreased Retinal Thickness

**FIGURE 3:**
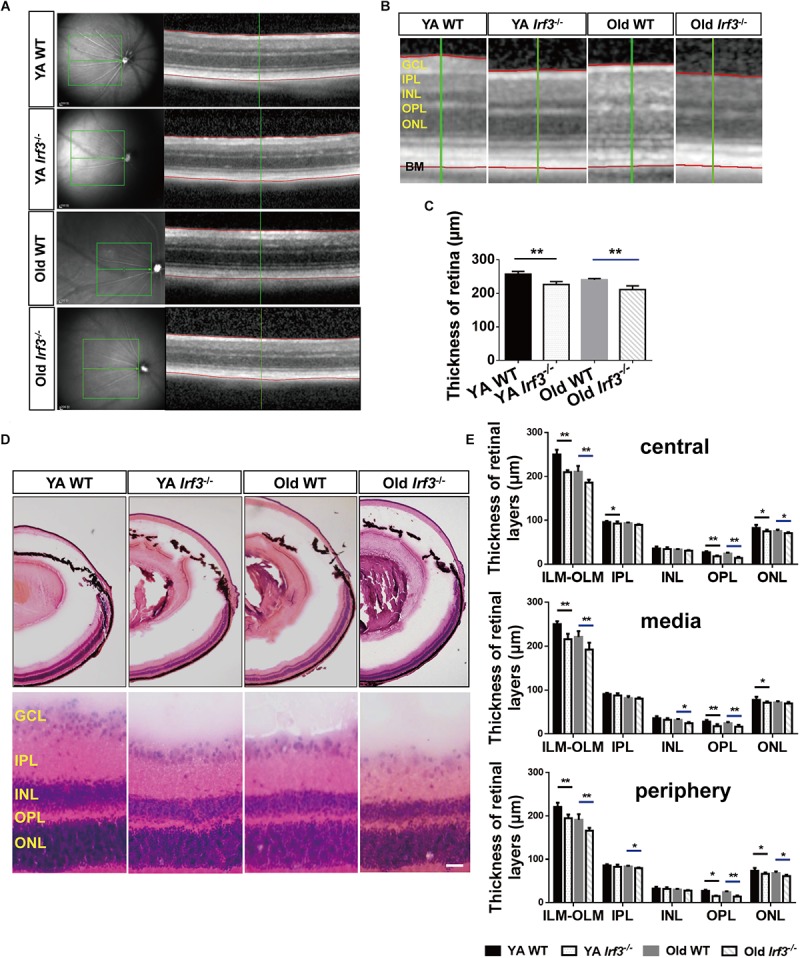
Deletion of *IRF3* interferes with retinal thickness. **(A)** Representative images of OCT scanning in both YA and old WT and *Irf3*^-/-^ mice. The green boxes indicate the examination area, which ranged from 250 (center) to 500 (periphery) μm from the optic nerve. The red lines indicate the edges of the inner limiting membrane (ILM) and basal membrane (BM), respectively. **(B)** The amplified picture in accordance with the area near the green line on the right side of **(A)**. ONL, outer nuclear layer; OPL, outer plexiform layer; INL, inner nuclear layer; IPL, inner plexiform layer; GCL, ganglion cell layer. **(C)** Quantification of the thickness of entire retina, from internal limiting membrane to BM, for each group, Bars represent the mean ± SEM (^∗∗^*P* < 0.01, *n* = 6 per group). **(D)** Representative graphs of HE retinal staining in YA WT, Old WT, YA *Irf3*^-/-^, and Old *Irf3*^-/-^ mice. The enlarged images were arranged in the second row. Scale bars = 30 μm **(E)** The quantification of thickness of designated retinal layers in three regions of the retina (central, media, periphery), including total thickness (from the internal limiting membrane (ILM) to the outer limiting membrane (OLM)), IPL, INL, OPL and ONL for each group (*n* = 6 per group). Data are given as mean ± SEM (^∗^*P* < 0.05, ^∗∗^*p* < 0.01).

To investigate the overall effect of *IRF3* deletion on retinal structure, retinal thickness in both the live and deceased mice was evaluated. As the OCT data showed, the thickness of the retina was significantly reduced in *Irf3*^-/-^ mice compared with WT mice of the same age (Figure 3A–C). To further understand which layer was responsible for the thickness change, we used HE staining. In agreement with the OCT result, the total thickness of three different retinal regions (central, media, periphery) was reduced in *Irf3* mutants (Figure 3D,E). Interestingly, regardless of the area in the retina, the thickness of the OPL and the outer nuclear layer (ONL) were both reduced in *Irf3*^-/-^ mice, and the OPL was remarkably reduced (Figure 3E). However, a reduction in retinal thickness did not appear in *Irf3*^-/-^ mice at the beginning or the end of the developmental window for photoreceptor synaptogenesis (P7, P21) (Supplementary Figures S2A,B). These results suggest that *IRF3* is essential for the structure of YA and Old retinas, especially the OPL.

### *IRF3* Deficiency Disrupts Synaptic Integrity in the Retina

**FIGURE 4:**
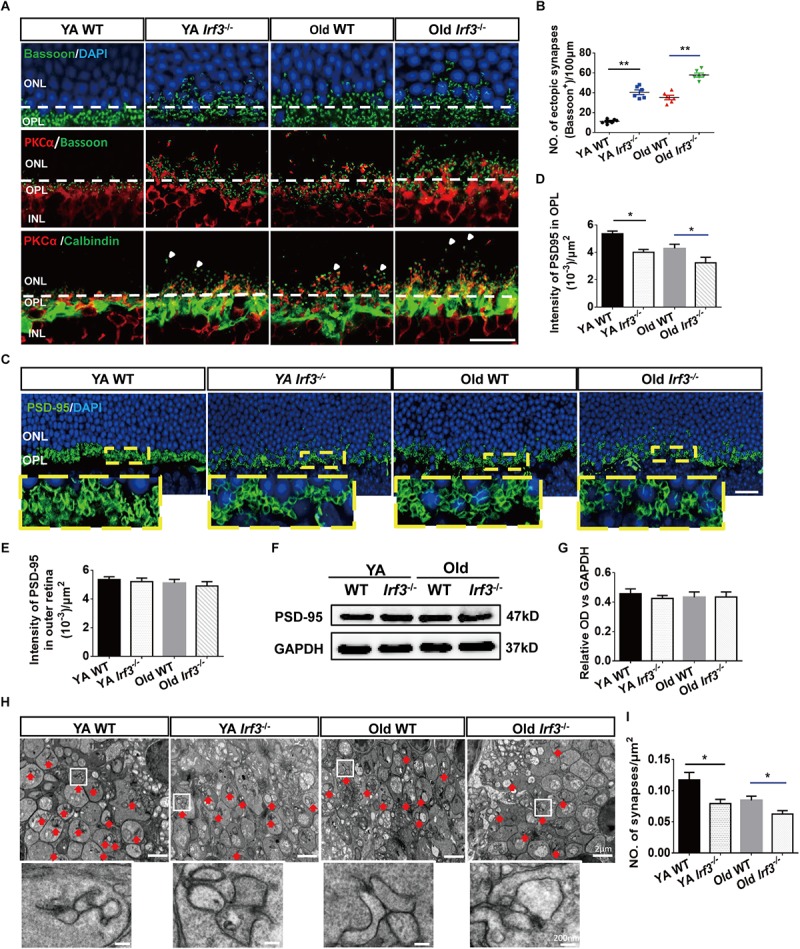
*IRF3* deficiency induces age-related synaptic changes in the retina. **(A)** Immunostaining for synaptic ribbon (bassoon, green), cell nuclear (DAPI, blue), rod bipolar cells (PKCα, red) and horizontal cells (calbindin, green). Ectopic pre-synapse (Bassoon) entered the ONL layer in YA and Old *Irf3*^-/-^ mice and old WT. Synaptic contacts between rod photoreceptors (Bassoon) and rod bipolar cell dendrites (PKCα, red) had the same changes in YA and Old *Irf3*^-/-^ mice and Old WT mice. Mislocation processes of rod bipolar and horizontal cells (arrows) were synchronously observed in YA and Old *Irf3*^-/-^ mice and Old WT mice. The white dotted line indicates the border of OPL/ONL. Scale bar = 20 μm. **(B)** The sprout synapses beyond the border (white dotted line) were quantified. Data are presented as mean ± SEM (^∗∗^*P* < 0.01, *n* = 6 per group). **(C)** Expression and localization of PSD-95 (photoreceptor synapse, green) in each group. Scale bars = 20 μm. **(D)** The quantification of average fluorescence intensity of PSD-95 in the OPL. Data are presented as mean ± SEM (^∗^*P* < 0.05, *n* = 4 per group). **(E)** The quantification of average fluorescence intensity of total PSD-95 in outer retina. Data are presented as mean ± SEM from 4 animals per group. **(F,G)** The levels of PSD-95 were not significantly altered in YA or Old *Irf3*^-/-^ mice or Old WT. Protein levels were assayed by immunoblot analysis **(F)**, quantified in **(G)**. Data are presented as mean ± SEM from 4 animals per group. **(H)** Representative electron microscope images show rod spherule synapses in the OPL of YA WT, Old WT, YA and Old *Irf3*^-/-^ mice (red arrow). Scale bar = 2 μm. White boxes are high magnifications of synaptic structures (Scale bar = 200 nm). **(I)** The quantification of the synapses density within the OPL of the retinas. Data are presented as mean ± SEM of 64 fields from 4 animals in each group (^∗^*P* < 0.05, ^∗∗^*P* < 0.01, *n* = 4 per group).

Based on the aforementioned structural and functional alteration, we sought to uncover the associated underlying pathological changes. As the thickness of the OPL is most clearly altered in *Irf3*^-/-^ retinas and photoreceptor cells and interneurons form synapses exclusively in the OPL (Hoon et al., 2014; Samuel et al., 2014), we investigated synaptic structure in the OPL by immunofluorescence staining. As shown by Bassoon and DAPI co-labeling, photoreceptor synaptic terminals (pre-synaptic) obviously withdraw into the ONL of the retina in *Irf3*^-/-^ mice compared with WT mice at both YA and Old stages (Figure 4A,B). Moreover, to identify synaptic contacts between photoreceptor cells (pre-synaptic) and dendritic terminals (post-synaptic) of rod bipolar cells in the OPL, we used double labeling with bassoon and PKCα. Both synaptic markers revealed that sprouts in YA and Old IRF3-deficient mice were dotted with numerous ectopic synapses, consistent with the location of Bassoon (Figure 4A). Similarly, the PKCα-positive dendrites of rod bipolar cells and calbindin-positive neurites of horizontal cells (the post-synaptic terminals) both extended further into the ONL in *Irf3*^-/-^ mice compared with WT mice (Figure 4A). Unexpectedly, Similar synaptic changes were not seen in *Irf3*^-/-^ mice at P7 and P21 (Supplementary Figure S2C).

In addition to the location alternation of the OPL synapse, we also found a reduced expression of PSD-95, a protein expressed on photoreceptor synaptic terminals (Koulen et al., 1998), in the OPL of retinas from the *Irf3*^-/-^ mice compared with WT mice of the same age (Figure 4C,D). However, the PSD-95 level was not significantly reduced in the outer retina of each group (Figure 4E–G), indicating that the decrease in synaptic density in the OPL was not caused by the change of the total number of synapses in the outer retina. To illustrate the ultrastructure of the synapses in the OPL, electron microscopy was performed. Triad structure and ribbon, which is typical of the morphology of rod spherule synapses in the OPL, was observed in all groups (Figure 4H, red arrow). Interestingly, the density of the triad structure was also remarkably reduced in *Irf3*^-/-^ mice compared with WT mice of the same age, which indicates that *IRF3* may be crucial for maintaining synapse density in the OPL (Figure 4H,I).

Based on our results, *IRF3* deletion results in the alternation of the normal architecture and density of synapse in the OPL. Collectively, the aged-related morphological and functional changes in *Irf3*^-/-^ mice are more obvious compared with WT mice (both the YA and Old groups), indicating *IRF3* deficiency may play a role in accelerating retinal senescence.

### Loss of *IRF3* Up-Regulates the Expression of Senescence Related Markers

**FIGURE 5:**
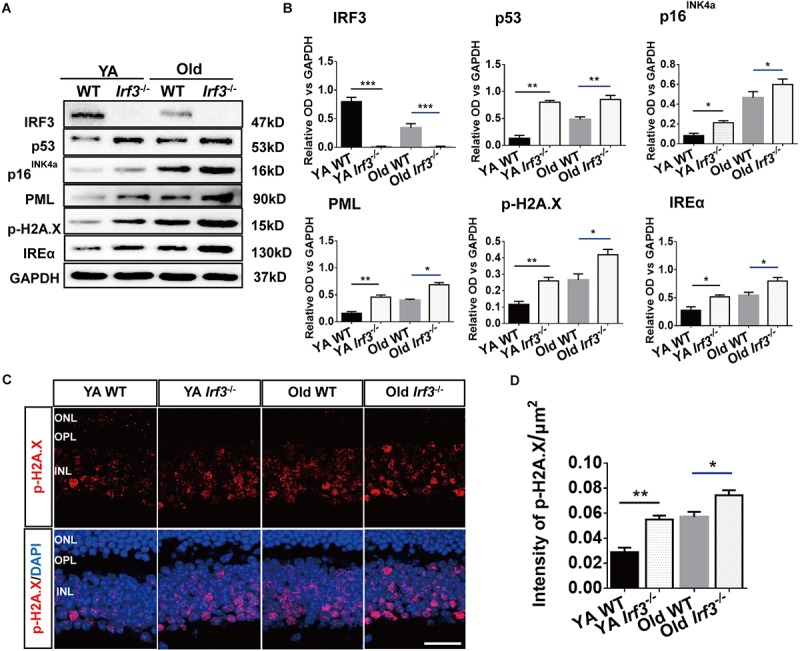
*IRF3* deficiency promotes the expression of senescence-related markers in retina. **(A)** Representative immunoblots show the expression of senescence markers (p53, p16^INK4a^, PML, IREα, p-H2A.X) and IRF3 in YA WT, Old WT, YA *Irf3*^-/-^ and Old *Irf3*^-/-^ mice. GAPDH was used as the loading control. **(B)** The quantification of *IRF3* protein expression and the senescence markers. Data are presented as mean ± SEM (^∗^*p* < 0.05, ^∗∗^*p* < 0.01, ^∗∗∗^*p* < 0.001, *n* = 6 per group). **(C)** Expression and localization of p-H2A.X in retinas of YA WT, Old WT, YA *Irf3*^-/-^ and Old *Irf3*^-/-^ mice. Scale bars = 30 μm. **(D)** The quantification of fluorescence intensity of p-H2A.X expression. Data are presented as mean ± SEM (^∗^*p* < 0.05, ^∗∗^*p* < 0.01, *n* = 5 per group).

Our results reveal a potential relationship between *IRF3* and senescence. To directly address this hypothesis, we took advantage of a series of senescence markers which classically reflect the stages of cellular senescence in retina. The protein level of p53, p16^INK4a^, inositol-requiring enzyme α (IREα), p-H2A.X and promyelocytic leukemia protein (PML) were evaluated in WT and *Irf3*^-/-^ retinas (Campisi and d’Adda di Fagagna, 2007; Oubaha et al., 2016; Yang et al., 2016). Qualitative and quantitative analysis indicated that *IRF3* deletion in the retina lead to higher expressions of these five markers than WT mice of the same age. Hardly any *IRF3* expression was detected in *Irf3*^-/-^ mice (Figure 5A,B and Supplementary Figure S3). In addition, dramatic reduced *IRF3* expression was found in Old WT mice compared with YA WT mice (Figure 5A,B, IRF3 panel and Supplementary Figure S3 , which implied that *IRF3* expression is affected during normal aging.

To further determine which neuronal cells of the retina might be involved in senescence, we applied immunofluorescence staining to p-H2A.X, a phosphorylated histone that appears in the nucleus of senescence cell (Fang et al., 2012; Yoshizawa et al., 2012). Our result revealed that p-H2A.X expressions were largely increased in *Irf3*^-/-^ mice compared with WT mice at both YA and Old stages (Figure 5C,D). Meanwhile, in *Irf3*^-/-^ retinas, p-H2A.X was mainly located in the INL (Figure 5C), indicating that some neuron cells of INL might be affected. Interestingly, by further co-localization of p-H2A.X with four types of cells in the INL of *Irf3*^-/-^ mice, we found that most of the p-H2A.X positive cells co-localized syntaxin I in the amacrine cells (Supplementary Figure S4). Together, these results indicate that the deletion of *IRF3* exacerbates the senescence of retina in YA and Old mice.

## Discussion

The Old retina often shows numerous functional and structural alternations, such as retinal dysfunction, decreased thickness, synaptic ectopia in the OPL, and a decreased number of photoreceptors, which provide some well-identified biomarkers for evaluating retinal aging (Eliasieh et al., 2007; Sullivan et al., 2007; Terzibasi et al., 2009; Nag and Wadhwa, 2012; Rodriguez-de la Rosa et al., 2012). However, the factors causing the alternations of retina during aging are poorly understood. In our present work, we found that *IRF3* expression was significantly decreased during aging and expressed in the OPL. *IRF3* deficiency in YA mice lead to functional and structural changes of the retina similar to those observed in old animals. Aging-related changes were also prominent in Old *Irf3*^-/-^ mice than Old WT mice. In particular, compared with WT of the same age, *IRF3*-deficient mice distinctly exhibited ectopic synapse from the OPL into the ONL, along with a decrease of the synapse density in the OPL. Moreover, the expression of several senescence markers was dramatically increased in the retina of *Irf3*^-/-^ mice. Besides, the absence of *IRF3* did not cause significant retinal abnormalities at P7 (photoreceptor synaptogenesis begins) and P21 (photoreceptor synaptogenesis completes fully) (Hoon et al., 2014) (Supplementary Figures S2A–C), implying that *IRF3* might not be necessary for early retinal development. Our results are the first to reveal that normal levels of *IRF3* are crucial for the structure and function of YA and Old retinas and that *IRF3* deficiency might contribute to accelerating retinal senescence for these age groups.

It has been previously reported that *IRF3* was related to age-related retinopathy (Kleinman et al., 2012). Moreover, its upstream regulatory factor (e.g., TLR3) and downstream target (e.g., IFN-β) are involved in retinal disease in old animals (Kumar et al., 2004; Hooks et al., 2008; Shiose et al., 2011; Luckoff et al., 2016). In our present study, the synthesis of *IRF3* was reduced in old animals under normal condition, indicating that *IRF3* might have relationship with retinal aging. Furthermore, we identified the location of *IRF3* via immunostaining and noticed that *IRF3* protein was mainly expressed at the synaptic terminals in OPL, which might affect synapse in outer retina.

Electroretinogram is one of the most efficient electrophysiological techniques for detecting retinal function (Perlman, 1995; Meigen, 2015). We explored the effect of *IRF3* deficiency on retinal function by ERG. In our experiments, *Irf3*^-/-^ mice showed a significant decrease in both a-wave and b-wave amplitudes (Figure 2A–C), which reflected functional deficits for both rod photoreceptor cells and interneurons. Indeed, ERG response recessions have been observed in some retinal degeneration mouse models (Chang et al., 2002). In most dystrophic mice, the loss of photoreceptors may be the principle reason for the decrease in ERG wave amplitudes of the retina (Cuenca et al., 2004, 2005; Barhoum et al., 2008). However, the *Irf3*^-/-^ mouse model might reveal not only a reduction in the number of rod photoreceptors but also significant synaptic alternations in the OPL, contributing to function deficits of ERG.

Reduced retinal thickness has been widely recognized in retinal senescence and other retinal degeneration processes (Archibald et al., 2011; Samuel et al., 2011; Rodriguez-de la Rosa et al., 2012). By combining OCT and HE staining methods, we consistently found that the aging process could cause a decrease of the entire retina thickness and also in some specific layers, such as the OPL (Figure 3A–E). Strikingly, *IRF3*-deficient YA and Old mice presented various degrees of retinal structure abnormalities reminiscent of the aging phenotype, both in live and deceased mice (Figure 3A–E). The OPL in the retina is characterized by the formation of synapse between photoreceptor cells and interneurons (Hoon et al., 2014). In this study, we found that *IRF3* deficiency caused a malposition of the synapse from the OPL to the ONL, accompanied by the loss of synapse density in the OPL (Figure 4A,C,E). Some previous studies found that relocation of synapse may be attributed to the changes of synaptic structure, reduced neurotransmitters releasing and retraction of axon terminals of photoreceptor cells (Brown et al., 1981; Jones et al., 2012). Our results showed that the expression of IRF3 was predominantly concentrated around the synapses of OPL (Figure 1C), implying that IRF3 might interact with synapses in the OPL. Meanwhile, synaptic ectopia and altered cell alignment in the ONL could lead to the OPL thickness change (Kaneko et al., 2011), so the synaptic rewiring in the *Irf3*^-/-^ mice (Figure 4A,C) may also result in the decrease of OPL thickness. To our knowledge, this is the first study to reveal that *IRF3*, which is a defined immune-related factor, could have an effect on retinal structure and function.

Until now, *IRF3* has mostly been known for regulating the expression of *IFN-β* and thus participating in a series of pathological progresses in retina (Halder et al., 2015; Luckoff et al., 2016), while our study addressed the potential role of *IRF3* in retina during the physical aging process. In our study, the lack of *IRF3*-induced structural and functional changes indicated senescence-like alternations. Importantly, in *IRF3*-deficient mice, the up-regulation of senescence-associated markers (e.g., p53, p16^INK4a^, PML, p-H2A.X, and IREα) further supported the role of *IRF3* in affecting the aging process (Figure 5A,B). Accordingly, Old mice that had a handful of *IRF3* level compared with YA mice also clearly showed increased level of classic senescence proteins in the physical condition. Given that it had been found that *IRF3* could be involved in senescence and premature aging in response to DNA damage (Yu et al., 2015), we took advantage of the senescence-associated DNA damage marker p-H2A.X to find the affected retinal neuron. Interestingly, p-H2A.X signals mainly appeared in INL of *Irf3*^-/-^ mice and Old WT mice, but not in the ONL. By further identifying the location p-H2A.X positive cells in *Irf3*^-/-^ mice, we found that the amacrine may be mostly influenced (Supplementary Figure S4). Since the *IRF3* are mainly expressed by bipolar cells, it is possible that the deletion of *IRF3* may indirectly impact other cells in the INL as reported before (Yoshizawa et al., 2012). In addition, the absence of *IRF3* may contribute to retinal changes through signaling pathways of these senescence markers (Kim et al., 2006; El Asmi et al., 2014; Diwaker et al., 2015), but further research is needed.

## Conclusion

Together, our study supports the idea that maintaining normal *IRF3* levels is important to the structural and functional normality of YA and Old retina. The absence of *IRF3* might be associated with the acceleration of retinal senescence. Future work with conditional *Irf3*^-/-^ mice targeting a precise cell in the retina would help identify the exact role that *IRF3* plays and the underlying mechanisms at work during this process. Our results uncovered a new function of *IRF3* in the retina and may provide meaningful insight into therapeutic strategies for age-related retinal degeneration diseases.

## Author Contributions

XZ designed and conducted the experiments, acquired and analyzed data, and wrote the manuscript. JZ acquired and analyzed the data. XC analyzed data and revised the manuscript. ZJ-Q helped with complementary experiments. XL, LL, HH, and WL conducted experiments and acquired data. JYe and SL designed the research studies, analyzed data and provided the funding. XZ and JYan conducted the research, analyzed data and provided the funding. All authors approved the final version of the manuscript.

## Conflict of Interest Statement

The authors declare that the research was conducted in the absence of any commercial or financial relationships that could be construed as a potential conflict of interest.
