# Supplementary material for: Interferon Regulatory Factor 3 Deficiency Induces Age-Related Alterations of the Retina in Young and Old Mice
Source: Front Cell Neurosci. 2019 Jun 20;13:272. doi: 10.3389/fncel.2019.00272 (PMC6596281; doi:10.3389/fncel.2019.00272)
Supplement: Supplementary file 1 [file Data_Sheet_1.docx]

**Supplementary Material**

**Supplementary Figures**


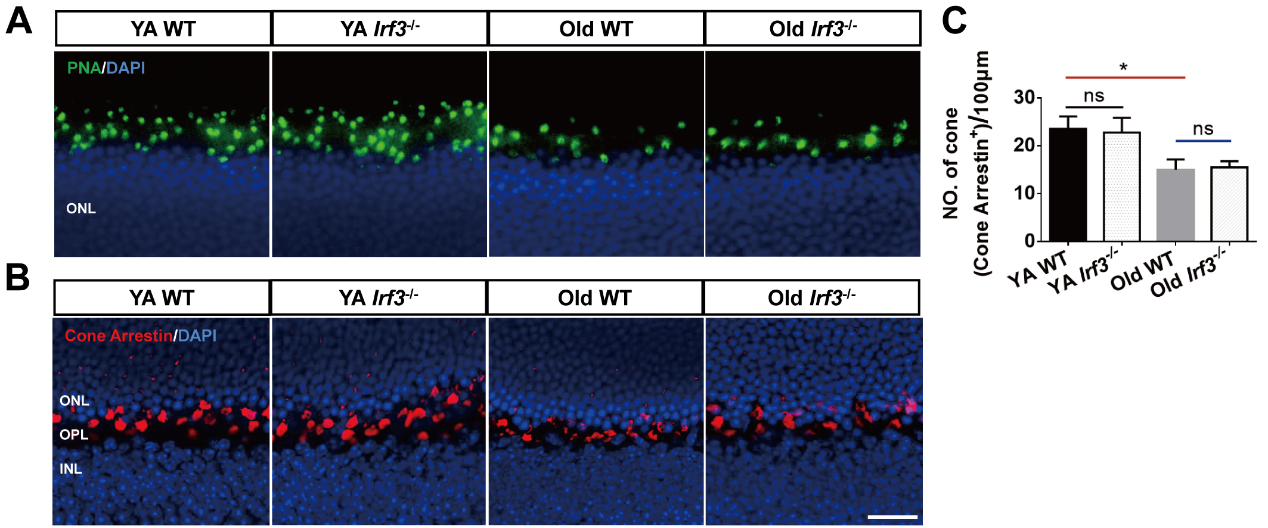


**Figure S1.** **(A-B)** Analysis of cone cells in WT and *Irf3*^-/-^ retina at both YA and Old stages. PNA (**A**, cone outer segment sheaths in green) and cone arrestin (**B**, cone pedicles in red) staining were normal in *Irf3*^-/-^ retina compared with that in WT retina of the same age. Nuclei in blue. Scale bar =20 µm. (**C**) Quantification of the number of cones per 100 µm in each group (cone arrestin). Data are presented as mean ± SEM (n=4 per group).


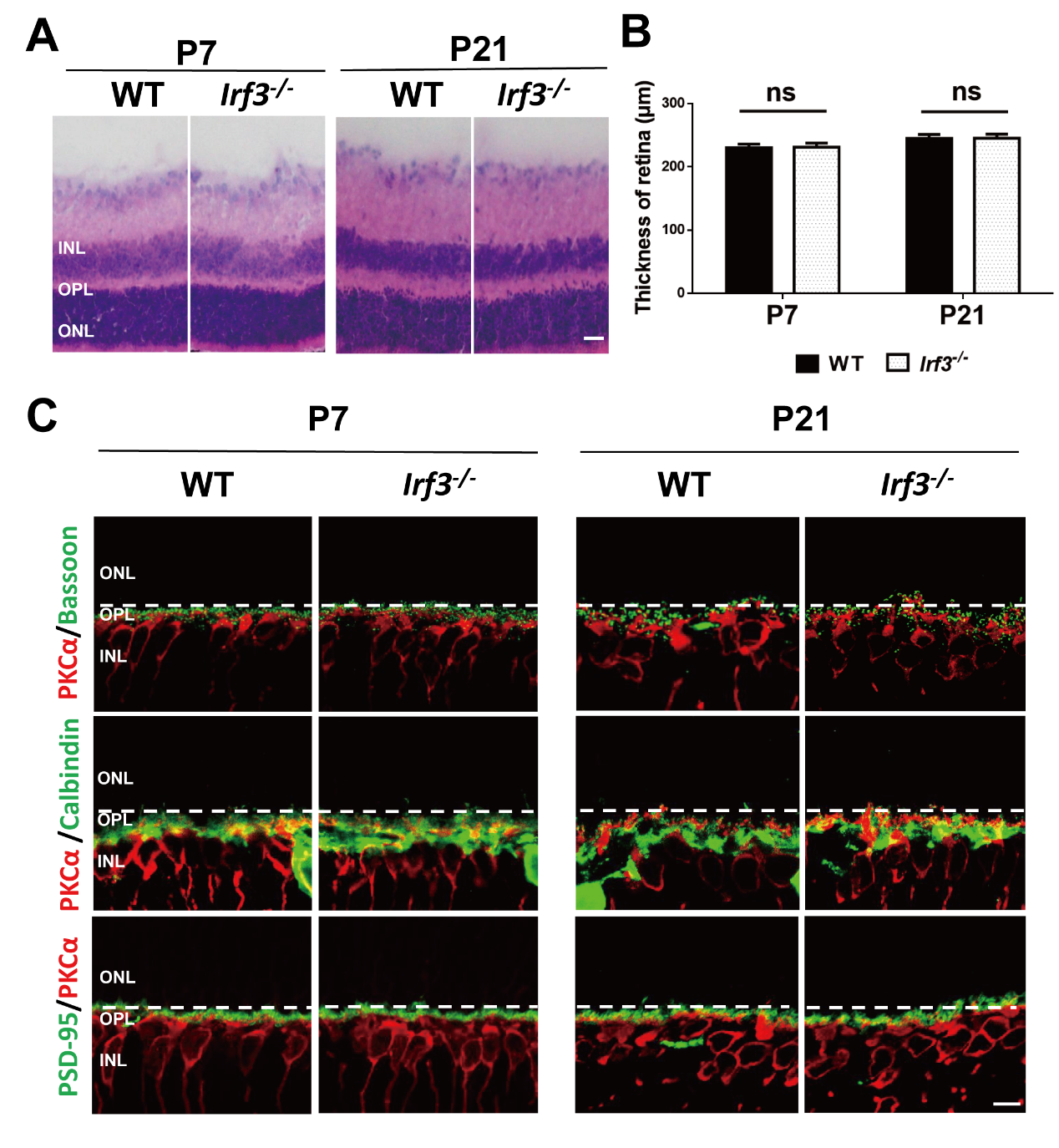


**Figure S2.** Morphological comparison of the WT and *Irf3*^-/-^ retinas at P7 and P21. (**A**) Analysis of retinal thickness in WT and *Irf3*^-/-^ retinas at P7 and P21 by HE staining. Scale bar =30 µm. (**B**) The quantification of the thickness of entire retina. Data are presented as mean ± SEM (n=4 per group). (**C**) A panel of synaptic markers was used to label retinal synapses from WT and *Irf3*^-/-^ mice at both YA and Old age. In each case, the synaptic markers labeled puncta located OPL. Bassoon (green), photoreceptor synapses; PKCα (green or red), rod bipolar cells; calbindin (green), horizontal cells; PSD95 (green), scaffolding protein in photoreceptor terminals. Scale bar =5 µm.


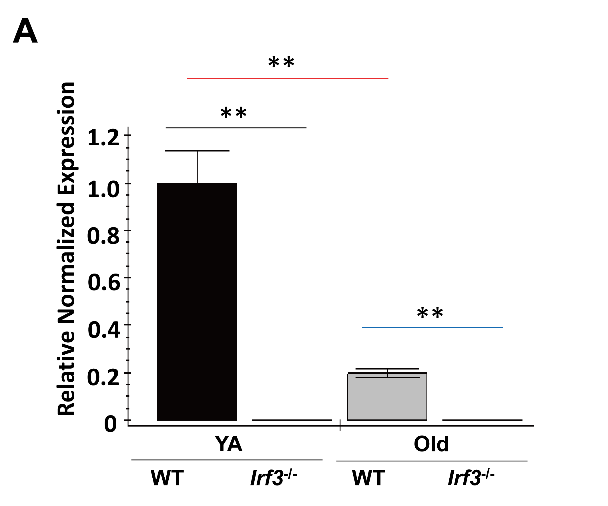


**Figure S3.** The mRNA expression of *IRF3* in WT and *Irf3*^-/-^ retina at YA and Old age. Data are presented as mean ± SEM (**p<0.01, n=4 per group).


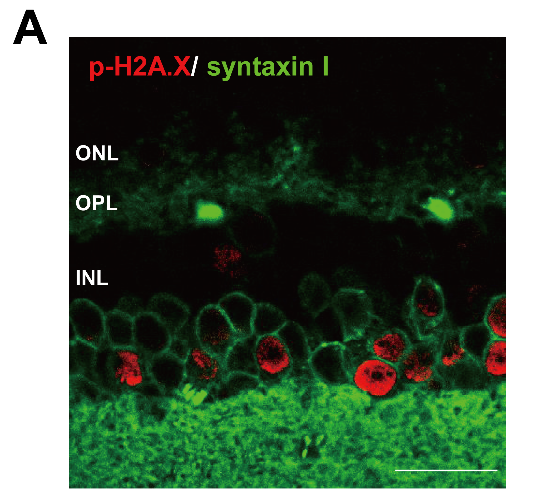


**Figure S4.** (**A**) Co-localization of p-H2A.X (red) and Syntaxin I (green) in *Irf3*^-/-^ retina; Syntaxin I , amacrine cell; Scale bar =20 µm.
